# The Role of Glucose-6-Phosphate Dehydrogenase in Skin Cancer Metabolism: A Paradigm Shift in Treatment Approaches

**DOI:** 10.3390/cancers17010048

**Published:** 2024-12-27

**Authors:** Anusha Abdullah, Jörg Kumbrink, Paris Liokatis, Andreas Mock, Ahdiya Abdullah, Ina Dewenter, Katharina Theresa Obermeier

**Affiliations:** 1 Department of Oral and Maxillofacial Surgery and Facial Plastic Surgery, Ludwig Maximilian University of Munich (LMU), 80337 Munich, Germany; 2 Institute of Pathology, Faculty of Medicine, Ludwig Maximilian University of Munich (LMU), 80337 Munich, Germany; 3 German Cancer Consortium (DKTK), Partner Site Munich, 80337 Munich, Germany

**Keywords:** skin cancer, glucose-6-phosphate dehydrogenase, melanoma, basal cell carcinoma, Merkel cell carcinoma, squamous cell carcinoma

## Abstract

Skin cancer is one of the most prevalent malignancies in the world, with increasing incidence. In 2022, the World Health Organization estimated over 1.5 million new diagnoses of skin malignancies, primarily affecting the older population. Surgical excision, particularly in the head and neck area, can cause aesthetic deficits and significantly impair patients’ quality of life. There are limited therapeutic options for advanced skin malignancies, and the development of resistance to targeted therapy further restricts treatment choices. Cancer metabolism may offer a novel approach to overcome these challenges. The pentose phosphate pathway, along with its rate-limiting enzyme, glucose-6-phosphate dehydrogenase, is essential for both the antioxidative response and the synthesis of ribonucleotides and may play a critical role in the proliferation and growth of cancer cells. This review examines current knowledge on the correlation between altered glucose-6-phosphate dehydrogenase expression and activity and skin cancer progression, with the aim of identifying a potential therapeutic target for treating advanced skin cancer.

## Simple Summary

Skin cancer ranks among the most prevalent malignancies globally. Current treatment for skin cancer involves surgery, chemotherapy, radiotherapy, and immunotherapy. Chemotherapy drugs have demonstrated resistance following several cycles, resulting in limited therapeutic possibilities for recurring and resistant cases. Targeting cancer metabolism to restrict cell growth may be a promising approach to increase the survival rates of skin cancer patients. One potential target is glucose-6-phosphate dehydrogenase, the rate-limiting enzyme of the pentose phosphate pathway. Inhibition of glucose-6-phosphate dehydrogenase activity could reduce cellular NADPH production, thereby increasing oxidative stress and limiting tumor progression.

## 1. Introduction

**Figure 1:**
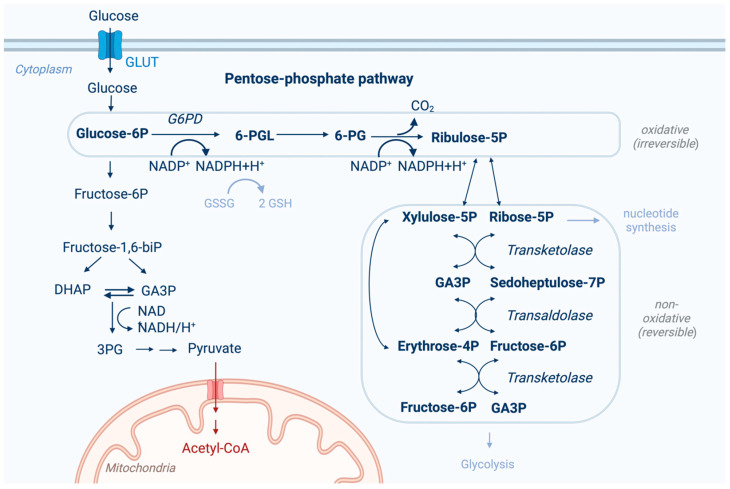
The pentose phosphate pathway, with an oxidative, irreversible, and non-oxidative, reversible arm branching from glycolysis. After the conversion of glucose to glucose-6-phosphate (Glucose-6P), the rate-limiting enzyme glucose-6-phosphate dehydrogenase (G6PD) oxidizes glucose-6P to 6-phosphogluconolactone (6-PGL) and reduces NADP^+^ to NADPH+H^+^. 6-PGL is hydrolyzed to 6-phosphogluconate (6-PG). 6-PG forms ribulose-5-phosphate (Ribulose-5P) by oxidative decarboxylation and generates NADPH+H^+^. NADPH can be used to regenerate glutathione (GSH) from glutathione disulfide (GSSG). Ribulose-5-phosphate is isomerized or epimerized to ribose-5-phosphate (Ribose-5P) or xylulose-5-phosphate (Xylulose-5P). The non-oxidative part of the pathway generates from ribose-5-phosphate and xylulose-5-phosphate through the transketolase reaction of sedoheptulose-5-phosphate (sedoheptulose-5P) and glyceraldehyde-3-phosphate (GA3P). These intermediates form fructose-6-phosphate (Fructose-6P) and erythrose-4-phosphate (Erythrose-4P) catalyzed by transaldolase. In the transketolase second reaction, a second fructose-6-phosphate molecule and glyceraldehyde-3-phosphate (GA3P) are generated and can be used in glycolysis. Adapted from the “Warburg Effect”, by BioRender.com (2024). Retrieved from https://app.biorender.com/biorender-templates (accessed on 24 November 2024).

The pentose phosphate pathway (PPP) diverges from glycolysis and oxidizes glucose-6-phosphate to produce NADPH (nicotinamide adenine dinucleotide phosphate) and ribonucleotides (Figure 1) [1]. It consists of two branches: oxidative and non-oxidative. The oxidative branch generates NADPH, which is essential for maintaining cellular redox balance and for the synthesis of ribonucleotides required for nucleic acid synthesis. In contrast, the non-oxidative branch produces pentose phosphates, which can be incorporated into DNA [1]. NADPH is critical for glutathione reduction and supports cell proliferation and antioxidant defense [2].

The rate-limiting enzyme of the PPP, glucose-6-phosphate dehydrogenase (G6PD), is crucial for cell growth, and its reduced activity impairs cell proliferation [1,3]. In cancer, aberrant activation of G6PD and the PPP has been observed, leading to increased NADPH production, fatty acid synthesis, and nucleic acid synthesis—processes that support rapid tumor growth and enhance resistance to oxidative stress [2,3].

The skin accounts for 15–17% of the total body mass and serves as the outer layer of the human body, making it easily susceptible to damage from pathogens, radiation, chemicals, and various other sources [4]. The primary risk factor for all skin cancer types is ultraviolet radiation (UVR); however, squamous cell carcinoma, in particular, can also arise in chronic lesions and wounds without UVR exposure [4]. Basal cell carcinoma is the most common form of skin cancer, followed by squamous cell carcinoma and melanoma [4]. Malignant melanoma is highly aggressive and responsible for nearly 80% of skin cancer-related deaths. Merkel cell carcinoma, a rare skin cancer associated with the Merkel cell polyomavirus, is characterized by high metastasis rates and mortality [4].

Skin cancer predominantly develops in areas frequently exposed to the sun, making the head and neck regions particularly susceptible to these malignancies. Treatment options for advanced skin cancer remain limited, and metastasized skin cancer—especially with resistance to therapies such as chemotherapy, targeted therapy and immunotherapy—is nearly incurable. In this review, we examine the role of G6PD in skin cancer metabolism, with the aim of identifying a novel therapeutic target for a multi-drug approach in the treatment of advanced skin cancer.

## 2. Modifications in the Metabolic Pathways Associated with Cancer

The hallmarks of cancer describe functionally essential features in cancer development, with the reprogramming of cellular metabolism to support enhanced cell proliferation, growth, and division being added in 2011. In the presence of oxygen, normal cells usually generate ATP via glucose and oxidative phosphorylation. However, cancer cells undergo a metabolic shift towards glycolysis, even in the presence of oxygen, a phenomenon known as the “Warburg effect” or “aerobic glycolysis” [5]. Although ATP generation via glycolysis is less efficient and requires higher glucose consumption, the glycolytic intermediates can be diverted into other pathways, such as the PPP, which generates nucleosides for DNA synthesis [5]. For instance, metabolic profiling reveals that melanoma cell lines exhibit the Warburg effect and consume more glucose than normal melanocytes [6,7].

Cancer cells also exhibit elevated levels of intracellular reactive oxygen species (ROS), which can enhance pro-oncogenic signaling pathways. Consequently, the upregulation of the PPP, with increased NADPH production, helps mitigate ROS levels and provides nucleotides for DNA synthesis and repair, thereby promoting tumor growth [1]. In this context, one of the key functions of G6PD in tumor growth may be protection from cell death. Increased levels of ROS in cancer cells, driven by accelerated metabolism and the hypoxic microenvironment, demand high levels of NADPH to prevent cell death [1].

Furthermore, abnormal expression of G6PD has been detected in various cancer types, including ovarian cancer, leukemia, lung cancer, gastric carcinoma, hepatocellular carcinoma, pancreatic carcinoma, and breast cancer [8]. Preclinical studies have demonstrated that silencing G6PD exerts antiproliferative effects on cancer cell growth [9,10,11]. A study by Li et al. (2023) showed that inhibition of G6PD in breast cancer cell lines reduced cell proliferation and increased oxidative stress. Inhibition of G6PD in mouse models also led to reduced tumor growth, suggesting that targeting G6PD may be a novel strategy for systemic breast cancer therapy [9].

NADPH is primarily synthesized through the oxidative PPP, while alternative mechanisms to generate NADPH are mediated by the activation of AMP-activated protein kinase (AMPK) [12,13]. On one hand, AMPK inhibits fatty acid synthesis, reducing NADPH consumption; on the other hand, it stimulates fatty acid oxidation, which increases NADPH levels through malic enzyme and isocitrate dehydrogenase 1 [12,13].

Inhibiting the oxidative PPP and the generation of NADPH through G6PD may trigger compensatory activation of fatty acid oxidation to provide cancer cells sufficient NADPH to survive redox stress. Further studies are needed to evaluate whether G6PD inhibition induces compensatory activation of alternative pathways.

These altered metabolic pathways represent potential therapeutic targets, offering new avenues for treatment.

## 3. The Role of G6PD on Cancer Metabolism, Progression, and Metastasis as Well as a Drug Target

Cancer cells adapt intracellular metabolic pathways to sustain accelerated proliferation and support the synthesis of essential cellular components, such as ribonucleotides and NADPH, for redox homeostasis. Upregulation of G6PD has been observed in various cancer types and is associated with advanced stage, metastasis, invasion, and poor survival outcomes [12,14,15].

**Figure 2:**
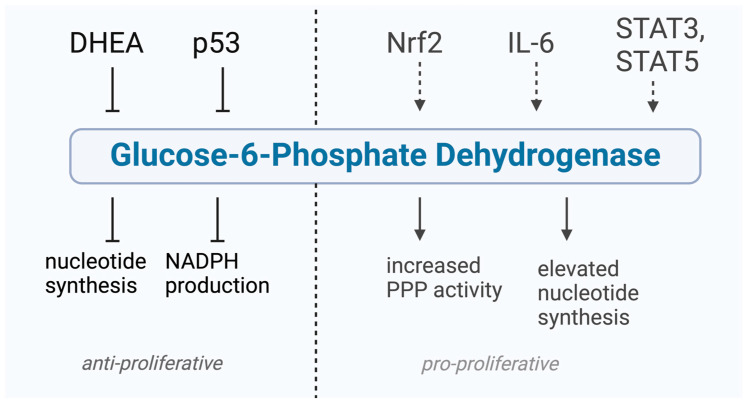
The G6PD inhibitor DHEA (dehydroepiandrosterone), the tumor suppressor p53, the transcription factors Nrf2 (nuclear factor erythroid 2-related factor 2), and STAT3, STAT 5 (signal transducer and activator of transcription) and the cytokine IL-6 (interleukin-6) have been linked to the activity of G6PD. Modulating the activity of this enzyme can influence cell proliferation, with inhibition resulting in reduced cell growth, while an increase in its activity may promote proliferation. Created with BioRender.com.

The role of G6PD on cancer progression, its potential as a drug target, and the influence of several factors on G6PD activity is further discussed in this section (Figure 2).

In 1965, Beaconsfield et al. demonstrated that a deficiency in G6PD decreases tumor incidence [16]. Other studies suggest that there is no clear evidence of a positive or negative correlation between G6PD deficiency and increased cancer incidence, making longitudinal studies to clarify whether G6PD deficiency is protective against cancer necessary [17].

Pearse et al. (1978) analyzed normal human skin chronically exposed to solar irradiation for two to six weeks and detected enhanced G6PD activity in the granular zone and increased G6PD epidermal activity [18]. These changes in enzyme activity were also observed in premalignant skin, implicating a role of G6PD in tumorigenesis [18].

Studies by Tang et al. (2021) suggest that for the malignant progression of HNSCC, nucleotide biosynthesis regulated by G6PD is more important than redox regulation, as the addition of ribose-5-phosphate significantly restored cell motility and invasiveness. In contrast, the addition of NADPH and GSH did not show similar effects [19].

A recent study by Wang et al. (2020) investigated how G6PD controls cancer metastasis. They analyzed G6PD expression in 105 patients with oral squamous cell carcinoma (OSCC) by immunohistochemistry and showed that high expression of G6PD was associated with a high lymphatic metastasis rate and poor prognosis [20].

Because the implications of G6PD on cancer incidence, progression, and metastasis have been studied and discussed for more than five decades, many approaches have been tried to limit its activity and thus prevent or reduce tumor progression.

The G6PD inhibitor dehydroepiandrosterone (DHEA) has demonstrated anti-tumor activity by repressing DNA synthesis and inhibiting G6PD uncompetitively [21]. Pashko et al. (1991) investigated the anti-tumor effects of a DHEA analog in a two-stage skin tumorigenesis model in mice [21]. Topical application of the synthetic DHEA analog (fluasterone; 16 alpha-fluoro-5-androsten-17-one), which does not have the sex-hormonal side-effects of DHEA, greatly inhibited 7,12-dimethylbenz(a)anthracene (DMBA), and 12-0-tetradecanoylphorbol-13-acetate (TPA) induced skin papilloma [22]. They suggest that by inhibiting G6PD, the synthesis of NADPH and ribose-5P for deoxyribonucleotide synthesis is reduced, leading to an inhibitory effect on carcinogenesis [21]. To validate the hypothesis that DHEA suppresses DNA synthesis by reducing ribonucleotide and deoxyribonucleotide synthesis, the four deoxyribonucleotides adenine, thymine, guanine, and cytosine were added to the culture medium of HeLa TCRC-2 cells, a human cervical carcinoma cell line, and reversed the fluasterone-induced inhibition of G6PD [22]. Moreover, topical application of fluasterone or corticosterone to mouse skin has shown anti-inflammatory and anti-hyperplastic effects [23]. The inhibition of G6PD partly mediates this effect and, therefore, the supply of NADPH-reducing equivalents and ribose-5P [23]. However, the production of oxygen-free radicals is inhibited by DHEA and may also contribute to its antiproliferative effects [22,24].

Furthermore, in vitro G6PD suppression by short-interfering (si)RNA or the inhibitor DHEA inhibited cell migration, invasion, and epithelial-mesenchymal transition of OSCC cells [20]. In an orthotopic xenograft model, lymphatic metastasis was reduced by inhibiting G6PD [20]. The authors propose G6PD as a therapeutic target for lymphatic metastasis of epithelial malignancies [20].

There is no single mechanism explaining DHEA’s anti-cancer effects since several hypotheses exist. The most generally accepted hypothesis is the inhibition of G6PD and, therefore, decreasing levels of ribose-5P and NADPH, which are necessary for cell growth [25]. In addition, DHEA also inhibits 3-hydroxy-3-methylglutaryl-CoA reductase, a rate-limiting enzyme in cholesterol biosynthesis, and inhibits DNA synthesis [25]. Other possible mechanisms contributing to the anti-neoplastic effects of DHEA are direct interference with the MAPK (mitogen-activated protein kinase) pathway and suppression of nitric oxide generation, which can cause oxidative damage [25].

Moreover, G6PD activity is influenced by various other molecules such as p53, Nrf2, IL-6, and STAT3/6.

The tumor suppressor p53 plays an essential role in regulating the PPP as it inhibits G6PD activity and thus the PPP, NADPH production and biosynthesis [26]. Mutated *TP53* genes are most prevalent in tumor cells, and it has been shown that inactivation of p53 via mutation or loss leads to enhanced G6PD and PPP activity as well as biosynthesis and NADPH production, resulting in tumor progression [26]. Therefore, G6PD inhibition might compensate for the effect of inactivated p53 on the PPP thereby limiting tumor malignancy.

Nuclear factor erythroid 2-related factor 2 (Nrf2) is a transcription factor that responds to oxidative stress and may play a critical role in carcinogenesis. Increased Nrf2 levels have been observed in head and neck squamous cell carcinoma (HNSCC) [19]. The oncogene c-MYC upregulates Nrf2 expression and drives the malignant progression of HNSCC [19]. It has been shown that activation of Nrf2 in xenograft tumor models induces tumorigenesis and promotes tumor growth and metastasis [19]. Notably, the expression of PPP genes was positively correlated with Nrf2 level, and elevated PPP activity was significantly associated with reduced patient survival, suggesting that it may serve as a predictor of poor overall survival [19]. The investigation indicates that Nrf2 modulates the PPP by increasing the protein levels and enzyme activity of G6PD and transketolase, thereby driving the malignant progression of HNSCC [19]. A combination of Cisplatin, the standard chemotherapy for HNSCC, with G6PD inhibitors such as DHEA, trans-polydatin, or 6-aminonicotinamide (6-AN), demonstrated synergistic cytotoxic effects [19].

Interleukin-6 (IL-6), an inflammatory cytokine, is frequently accumulated in the tumor microenvironment [27]. IL-6 activates Janus kinases (JAK), which, in turn, trigger the nuclear translocation of the transcription factor STAT3 (signal transducer and activator of transcription 3). Once in the nucleus, STAT3 influences the transcription of target genes that promote tumor cell proliferation, survival, and metastasis [27]. The impact of IL-6 on the metabolic flux of the PPP has been explored in several studies [28]. In human tongue squamous cell carcinoma lines CAL-27 and HSC-3, IL-6 treatment resulted in enhanced nucleotide synthesis and increased G6PD activity, thereby activating the PPP, although G6PD protein expression remained unchanged [28]. Further investigation revealed that IL-6 induces JAK2-mediated phosphorylation of G6PD, increasing its activity by more than 10-fold through the alleviation of its binding with glucose-6-phosphate [28]. Moreover, their results suggest that JAK2-dependent G6PD phosphorylation is essential for IL-6-induced nucleotide synthesis and thus plays a crucial role in OSCC cell proliferation and tumor growth [28]. By disrupting these events and inhibiting G6PD, tumor growth may be effectively limited.

Additionally, G6PD activity appears to regulate the phosphorylation of STAT proteins. STAT3 and STAT5 promote the expression of pro-survival and pro-growth genes and are constitutively active in various cancers [3,29]. In melanoma, highly activated STAT3 and STAT5, along with a potential role of G6PD in cancer mediated by these STAT proteins, has been observed [30].

## 4. Current Treatment Perspectives for Skin Cancer

The standard therapeutic approach for treating skin malignancies is surgical excision with safety margins [31,32]. However, this can lead to unsatisfactory aesthetic outcomes, particularly in the head and neck area, highlighting the need for alternative therapeutic options. Nonsurgical management of skin cancer, targeting the neoplastic tissue directly, can significantly reduce morbidity and improve the quality of life for patients [31]. Metastasized skin cancer is still considered largely incurable, underscoring the urgent need for the development of novel treatment options for these cases.

For melanoma and Merkel cell carcinoma (MCC) patients, surgical treatment with wide excision is often insufficient, as these cancers metastasize rapidly [4]. Approximately half of melanoma patients exhibit the BRAF V600E mutation, which can be targeted by therapies that inhibit the mitogen-activated protein kinase (MAPK) pathway, leading to significantly improved overall survival [4]. However, targeted therapies and immunotherapies, as well as radio- and chemotherapy for metastatic melanoma, all have one limitation: melanoma cells often develop resistance after only a few cycles of treatment [4]. Additionally, there are currently no effective therapies for patients with metastatic melanoma who lack the BRAF V600E mutation or do not respond to immunotherapy, making interference with the PPP in these melanoma cells a possible area for therapeutic exploration.

Tumor heterogeneity can lead to resistance to targeted therapies and contribute to disease progression [33]. However, there are limited treatment options for patients undergoing targeted therapy who develop resistance, highlighting the urgent need for new drugs in the treatment of metastatic melanoma [33]. Recently, the combination of BRAF inhibitors, such as vemurafenib and dabrafenib, with the MEK inhibitor trametinib has been shown to prolong response rate and delay the onset of resistance [34]. Despite this, drug resistance persists, as mutations in MEK1/2 can reactivate the MAPK pathway [34]. A multi-drug approach that includes inhibitors of cellular metabolism, such as those targeting the PPP, may offer additional benefits for patients.

Menzies et al. (2014) proposed a combination of MAPK inhibitors with other pathway inhibitors targeting the cell cycle, the PIK3 (phosphoinositide 3-kinase) pathway, and epigenetic factors to inhibit melanoma cell function [34].

Combining G6PD inhibitors with BRAF and MEK inhibitors could potentially slow the onset of resistance. A neoadjuvant approach aimed at prolonging the efficacy of targeted therapies before resistance develops may be a promising strategy for extending overall survival.

Table 1 presents a summary of established therapeutic targets and conventional chemotherapy agents used in the treatment of skin cancer.

### 4.1. Melanoma: Treatment Strategies and the Role of G6PD in Therapeutic Approaches

Malignant melanoma is one of the most aggressive forms of skin cancer and accounts for the majority of skin cancer-related deaths [4]. Due to its complex heterogeneity, the cellular and molecular mechanisms underlying melanoma are not fully understood, and this complexity might contribute to resistance to therapies [40,41,42]. Treatment guidelines and 5-year survival rates for malignant melanoma are summarized in Table 2.

In many patients with metastatic melanoma, mutations in the BRAF oncogene, which enhance oncogenic signaling through MAPK activation, are frequently observed [45]. Over 50% of melanoma patients harbor BRAF mutations, with 70–90% of these being the V600E mutant [45,46]. Vemurafenib is used to treat BRAF V600-mutated melanoma, but resistance often develops over time, partly through the upregulation of the PPP [47]. While targeted therapy improves overall survival, resistance to BRAF inhibitors and disease progression typically occur after a few years of treatment [48]. Khamari et al. (2018) investigated how melanoma cells resistant to BRAF inhibitors survive under oxidative stress. Their findings revealed that BRAF inhibitor-resistant cells exhibit upregulated PPP genes and are able to survive under oxidative stress due to elevated glutathione levels [48]. Targeting glutathione synthesis or regeneration, which relies on NADPH produced via the PPP, could potentially slow the onset of resistance. This hypothesis is further supported by studies showing that Nrf2, which is upregulated in BRAF inhibitor-resistant melanoma cells, plays a crucial role in glutathione recycling [48]. Moreover, Nrf2 regulates the antioxidant response and protects against UVR-induced oxidative stress [49]. However, high levels of Nrf2 have also been correlated with poor survival and invasive growth in melanoma [50].

Moreover, reversing the Vemurafenib-induced upregulation of the PPP by Norcantharidin, an anti-cancer drug, showed an inhibitory effect on proliferation and may be helpful in the development of new therapeutic strategies for melanoma patients with acquired resistance to standard therapy [47].

The role of G6PD as an antioxidant in melanoma cells A375 has been studied, and the inhibition of G6PD by G6PD knockdown sensitized melanoma cells to oxidative stress, decreased proliferation, and enhanced apoptosis [51]. Many studies suggest a central role for the PPP as an essential redox metabolic pathway contributing to malignant melanoma progression [49]. In this context, histochemical investigations of 40 primary tumors of malignant melanoma revealed a strong enzyme reaction of G6PD in superficial spreading and nodular melanoma, suggesting a high DNA synthesis rate [52]. Lentigo maligna melanoma cells showed a weaker reaction of G6PD and, therefore, a lower DNA synthesis rate, which clinically correlates with the slower growth rate of lentigo maligna melanoma compared to other types of malignant melanoma [52]. Daily cortisone injections resulted in 58% inhibition of melanoma growth in mice and decreased G6PD enzyme activity, suggesting that G6PD may be essential for tumor growth [53]. Today, there are more effective anticancer drugs available, and the use of steroids in oncology is now limited to managing the side effects of immunotherapy.

Yurchenko et al. investigated the effect of suppressing the expression of G6PD with antisense oligonucleotides on cutaneous melanoma in mice. Melanoma cells were injected subcutaneously, and the tumor size was measured. A reduction in tumor size by 57% compared to the control group was observed, though after discontinuing the treatment, the tumor grew rapidly. Furthermore, an increase in apoptosis and a significant decrease in the cell proliferation index of melanoma cells were detected. Oligonucleotides demonstrated mild toxicity due to interactions with plasma proteins. They can lead to elevated serum transaminases, thrombocytopenia, and complement cascade activation, which usually occur at higher doses than clinically applied. However, the authors only recommend using this therapy locally to prevent the development of metastases because suppressing G6PD systemically may lead to severe side effects [54]. Using low-dose inhibitors in a multi-drug approach in synergy with chemotherapy or targeted therapy could reduce the toxicity of systemically applied G6PD inhibitors and enhance the effect of the other drugs.

Also, the relationship between G6PD and cell proliferation and apoptosis was investigated in a melanoma mouse model [30]. A375 human melanoma cells with knocked-down G6PD gene expression were injected intradermally into the mice, and tumor growth and mouse survival were measured [30]. Tumor sizes were the smallest in the group with G6PD deficiency; accordingly, the most extensive tumors were observed in human dermal melanoma cells with the G6PD wild type, in which the highest G6PD activity was detected [30]. The expression and activity of G6PD positively correlated with melanoma weight, growth, and differentiation, suggesting that G6PD may play an important role in tumor malignancy [30]. The upregulation of the cell cycle proteins cyclin D1 and E in the group with high levels of G6PD indicates that melanoma cells may survive and proliferate more effectively by regulating the cell cycle [30].

Furthermore, a positive correlation between mRNA and protein expression of the cell apoptosis-inhibiting factors Bcl-2 and Bcl-xl was observed, suggesting that cell apoptosis in melanoma may be regulated by G6PD [30]. G6PD silencing leads to the downregulation of the positive cell cycle regulators, such as cyclin D1 and CDK5, while upregulating negative regulators p53 and p21. This resulted in cell cycle arrest at the G1/S checkpoint, effectively inhibiting melanoma cell proliferation [3,55].

The combination of the G6PD inhibitor 6-AN and Metformin, a first-line oral treatment for type 2 diabetes, was found to reduce melanoma cell line growth [56]. Notably, inhibiting G6PD in eight melanoma cell lines enhanced the effect of Metformin, leading to increased apoptosis and reduced cell growth and survival [56]. However, when Metformin was administered as monotherapy, it demonstrated only limited efficacy in treating melanoma [57]. These findings suggest that new therapeutic strategies could be developed by combining G6PD inhibition with other anticancer agents to enhance or potentiate their antitumoral effects.

Nakamura et al. (2024) demonstrated reduced tumor growth in G6PD-knockdown malignant melanoma cells by decreasing NADPH production and reducing oxidative stress tolerance. They propose that targeting the PPP could induce immunogenic cell death. Furthermore, their findings suggest that inhibiting G6PD in part of the tumor enhances the sensitivity of other lesions to immunotherapy [58]. The authors advocate for the local injection of G6PD inhibitors in combination with immunotherapy [58].

Numerous studies have highlighted the pivotal role of metabolic reprogramming and altered PPP activity in melanoma progression. This is particularly relevant for advanced tumors resistant to targeted therapies and immunotherapies. Targeting the altered metabolism of melanoma cells, which drives their growth, may offer a promising therapeutic approach. The use of G6PD inhibitors as adjuncts to targeted therapy and/or immunotherapy could potentially delay the onset of resistance, thereby extending survival in patients with advanced, metastatic melanoma.

### 4.2. Non-Melanoma Skin Cancer: Treatment Options and the Role of G6PD

Basal cell carcinoma (BCC) and squamous cell carcinoma (SCC) are the most common types of non-melanoma skin cancers [4]. Compared to melanoma, SCC and BCC generally have a better prognosis and can be effectively treated through surgical excision [4]. However, the size of the excision margins required often leads to aesthetic deformation, particularly in the face. While metastasis is less frequent than in melanoma, it still can occur, particularly in older patients with multiple comorbidities. For such cases, non-invasive treatment options are crucial.

Although research on the PPP in non-melanoma skin cancer is limited, increased activity of G6PD has been observed in human SCC, carcinomas in situ, and hyperkeratotic lesions with dysplasia [59]. For instance, Elias et al. (1980) reported a case of a 31-year-old woman with metastatic BCC, initially treated nine years prior. Histopathological analysis revealed elevated activity of both G6PD and 6-phosphogluconate dehydrogenase, key enzymes of the PPP [60].

Retinoids like etretinate have teratogenic potential, limiting their clinical use to keratinization disorders, severe psoriasis, and T-cell lymphoma [61]. However, they have also been described to have a suppressive effect on skin carcinogenesis [61]. Patients with solar keratoses and BCC have been treated with the etretinate and followed for six to 18 months [62]. During this treatment, G6PD activity was measured in biopsies taken at various timepoints. A reduction in G6PD activity was observed across the epidermis, from the granular to the basal cell layer in the treated skin, while no changes were detected in the uninvolved skin. Correspondingly, the size and number of lesions significantly decreased during treatment [62]. This suggests a correlation between tumor growth and G6PD activity in BCC.

As a result, a link between tumor growth and G6PD activity has been observed, and studies indicate a positive correlation. However, no studies to date have investigated the direct impact of inhibiting G6PD on tumor growth in BCC and SCC. Further research is needed to determine whether G6PD inhibitors could be used in the chemotherapy of these malignancies.

Similarly, MCC, a rare malignant skin cancer with high recurrence and metastasis rates, remains understudied in the context of G6PD inhibition (Table 3) [63]. Cases of spontaneous regression and lymph node lesions without skin involvement have been reported [63].

Nakamura et al. (2020) described a positive correlation between high expression of programmed death ligand 1 (PD-L1)—a molecule that suppresses T-cell activation—and better prognosis [66]. However, due to the heterogeneity of PD-L1 expression, its potential as a predictive biomarker remains limited [67]. In their study, the authors performed immunohistochemical analyses on 90 samples from patients with histologically diagnosed MCC and RNA sequencing on 41 samples. They also measured G6PD activity in blood samples from MCC patients. Several patient groups were identified based on G6PD expression, which reflects potential immune resistance, and PD-L1 expression, indicating the strength of the immune response [63]. G6PD expression was significantly regulated in the “cell division type” and was found to positively correlate with metastasis and negatively correlate with PD-L1 expression [67]. The authors speculate that low expression of G6PD is associated with high immune activity, suggesting a better response for immune checkpoint blockade therapy. They also demonstrated the potential of G6PD as a prognostic biomarker. Approximately half of MCC patients treated with immune checkpoint inhibitors, such as the PD-L1 inhibitor Pembrolizumab, do not respond to treatment; G6PD blockade could be a promising strategy for these patients [63].

Nakamura et al. (2020) hypothesize that G6PD blockade could induce immunogenic cell death, but since it also suppresses immune cell activity, tumor-selective G6PD inhibition will be necessary [67,68]. The challenge, therefore, lies in developing a mechanism to selectively suppress G6PD in tumors.

## 5. Future Directions

G6PD plays a crucial role in cellular antioxidant defenses by producing NADPH and modulating the intracellular redox potential [69]. Although cancer cells that overexpress G6PD are vulnerable to ROS accumulation, normal cells are also at risk. Tissues with high metabolic activity, particularly those sensitive to oxidative damage, may be adversely affected by G6PD inhibition and the consequent reduction in NADPH synthesis. For example, red blood cells depend on G6PD for protection against oxidative damage, and hereditary G6PD deficiency leads to hemolytic anemia due to the generation of free radicals [2,70]. Whether inhibiting G6PD as part of cancer treatment could trigger hemolytic anemia and result in severe side effects remains to be determined.

Polydatin, an inhibitor of G6PD, has been shown to inhibit cancer cell growth and reduce lymph node metastasis in HNSCC cell lines and an experimental orthotopic model of oral cancer [10]. Furthermore, a synergistic effect has been observed when Polydatin is combined with Cisplatin, enhancing the cytotoxicity against cancer cells [10]. Phase II clinical trials demonstrated no significant cardiovascular, hepatic, bone marrow, or renal toxic effects when Polydatin was administered twice daily for three months [10,71]. Similarly, RRx-001, another G6PD inhibitor, has been evaluated in phase III clinical trials involving patients with refractory solid tumors, with no evidence of hematological toxicity [2,72]. The application of these agents in clinical trials for the treatment of advanced melanoma may provide valuable insights into the efficacy and potential benefits of G6PD inhibitors.

Aurora et al. (2022) mutated G6PD in patient-derived melanomas and demonstrated that reduced G6PD function in these melanomas is associated with increased ROS levels, decreased NADPH, depleted glutathione, and reduced spontaneous metastasis compared to control melanomas [15]. The increase in oxidative stress was compensated for by higher activity of malic enzyme and enhanced glutaminolysis, where glutamine is converted into pyruvate, with the production of NADPH by malic enzyme [15]. Additionally, they showed that during metastasis, melanoma cells become more dependent on G6PD, likely due to increased oxidative stress associated with metastasis [15]. Melanoma cells appear to possess multiple protective mechanisms against oxidative stress, suggesting that compensatory pathways for managing oxidative stress may also play a role. Further investigation is required to evaluate how these compensatory mechanisms could influence the effectiveness of G6PD inhibitors in cancer treatment.

**Figure 3:**
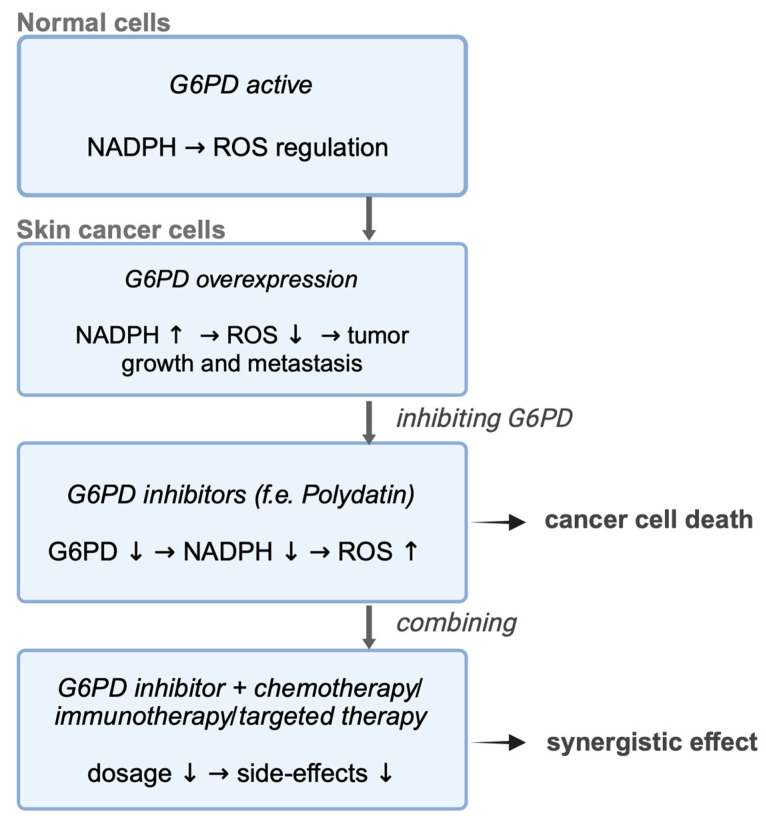
A schematic representation of the potential mechanism underlying G6PD inhibition and its therapeutic implications. Inhibition of G6PD results in reduced NADPH levels, leading to ROS accumulation, which induces oxidative stress and causes cell death. The combination of G6PD inhibitors with conventional chemotherapy, immunotherapy, or targeted therapy may reduce the required drug dosage, thereby minimizing systemic toxicity and side effects. Created in https://BioRender.com.

Figure 3 provides an overview of the potential role of G6PD in skin cancer development.

## 6. Conclusions

The PPP plays a crucial role in cancer development, with G6PD overexpression linked to the progression of various malignancies. This overexpression promotes cancer cell proliferation, tumor growth, and metastasis by reducing ROS and facilitating ribonucleotide synthesis. In skin cancers, G6PD upregulation is frequently observed in primary tumors and metastases, positioning it as a potential target for disrupting cancer metabolism. While targeting metabolic pathways such as folate metabolism has shown promise in anti-cancer therapy, drugs that target cellular metabolism can cause toxicity to normal cells and severe side effects.

Combining multiple therapeutic targets can reduce the required dose of chemotherapeutic agents, enhance the effectiveness of immunotherapy, and minimize adverse effects. Identifying specific metabolic pathways on which cancer cells rely may provide treatment options with minimal impact on normal cells [73]. Inhibiting the PPP, particularly its rate-limiting enzyme G6PD and its modulators like Nrf2, presents a challenge but holds considerable promise for cancer treatment.

We examine the potential roles of G6PD inhibition in the treatment of skin cancer. G6PD inhibition in skin cancer therapy could involve low-dose chemotherapy combinations, offering synergistic effects with reduced side effects. Targeted therapy, when combined with G6PD inhibitors, may help overcome resistance in malignant melanoma. A neoadjuvant approach utilizing G6PD inhibitors could reduce tumor size, improve surgical outcomes, and decrease the risk of metastasis. However, further evaluation is necessary to develop these strategies. This review aims to promote further investigation into targeting the metabolic pathways of skin cancer and to explore innovative therapeutic options for advanced skin malignancies.

## Figures and Tables

**Table 1:** An overview of key therapeutic targets and conventional chemotherapy agents utilized in the management of skin cancer.

	Targeted Therapy	Immunotherapy	Chemotherapy
Melanoma [35,36]	BRAF V600E/K MEK 1/2 NTRK c-KIT	Anti-CTLA-4 Anti-PD-1/PD-L1 IL-2	Decarbazine Temozolomide
Squamous cell Carcinoma [37]	EGFR	Anti-PD-1/PD-L1	Platinum-based drugs 5-Fluorouracil Bleomycin
Basal cell carcinoma [38]	Hedgehog	Anti-PD-1	5-Fluorouracil Platinum-based drugs
Merkel cell carcinoma [39]		Anti PD-1/PD-L1	Platinum-based drugs Etoposide Taxane Anthracyclin

**Table 2:** Current standard treatment of malignant melanoma according to the AJCC (American Joint Committee on Cancer) classification from 2016 [36].

Stage	Standard Treatment	5-Year Survival Rate [43]
0 (melanoma in situ)	Excision, Mohs surgery	100%
I	Excision, SLNB (Sentinel Lymph Node Biopsy). Cancer cells in lymph nodes: lymph node dissection, immune checkpoint inhibitors (pembrolizumab), targeted therapies (BRAF mutation)	98%
II	Excision, SLNB. Cancer cells in lymph nodes: lymph node dissection, immune checkpoint inhibitors (pembrolizumab), targeted therapies (BRAF mutation)	90%
III	Excision (if resectable), lymph node dissection, adjuvant immunotherapy, targeted therapy, radiation therapy, injection of T-VEC vaccine, interleukin-2, Bacille Calmette-Guerin (BCG) vaccine	77%
IV (metastasized)	Excision (if resectable), immunotherapy, targeted therapy, chemotherapy	29.8% [44]
recurrent	Immunotherapy, targeted therapy, chemotherapy, palliative therapy	

**Table 3:** Current standard treatment of MCC according to the AJCC (American Joint Committee on Cancer) classification from the 8th edition and survival rates in the different stages of MCC [64,65].

Stage (AJCC)	Treatment	5-Year Survival Rate [65]
Local (stage I, II)	Surgery, SLNB, radiotherapy	51%
Regional (stage III)	Surgery, radiotherapy, immunotherapy when not resectable (Pembrolizumab), chemotherapy and immunotherapy in clinical trials	35%
Disseminated (stage IV)	Immunotherapy (Avelumab, Pembrolizumab), immunotherapy in clinical trials (Nivolumab, Ipilimumab), chemotherapy, surgery as palliative treatment	14%

## Data Availability

The data used to support the content of this study are included within the article.
